# Designing a Clinical Pharmacy Primary Care Intervention for Myocardial Infarction Patients Using a Patient and Public Involvement Discussion

**DOI:** 10.3390/pharmacy8010013

**Published:** 2020-01-24

**Authors:** Zahraa Jalal, Vibhu Paudyal, Shahad Al-Arkee, Gillian Dyson, John Marriott

**Affiliations:** 1 School of Pharmacy, College of Medical and Dental Sciences, University of Birmingham, Birmingham B15 2TT, UK; v.paudyal@bham.ac.uk (V.P.); S.K.K.Al-arkee@pgr.bham.ac.uk (S.A.-A.); J.F.Marriott@bham.ac.uk (J.M.); 2 Patient and Public Involvement in Research Representative UK, Birmingham B15 2TT, UK; gilliandyson@hotmail.co.uk

**Keywords:** clinical pharmacists, clinical pharmacy services, patient and public involvement, myocardial infarction, discharge, patient

## Abstract

**Objective**: to conduct a Patient and Public Involvement (PPI) focus group session. To help inform the design of a clinical pharmacy intervention in primary care for patients after a coronary event. **Methods**: this study followed a public involvement method. Community members of the public and community engaged research patients who had experienced myocardial infarction where invited to actively take part in a focus group discussion. This is to share past experiences and provide input and advice into the design of a potential research proposal. The session took place at a cardiac rehabilitation centre. **Results**: four key themes were identified from the focus group these included: experiences with pharmacy and primary care services, medicines knowledge, the pharmacist role and building rapport with healthcare professionals. Nine participants and three researchers attended the PPI discussion session. Seven of the participants were patients who had experienced a cardiac event in the last three months and two were carers. Primary care pharmacy services both clinical and public health were not very familiar to the participants. Different experiences with clinical pharmacy services were reported by participants, while one experience was reported to be helpful others perceived community pharmacists to be to be busy and isolated behind a counter. A general practice GP based specialist nurse was a familiar model of care unlike a specialist clinical pharmacist GP based care role. Participants reported limited time in GP consultations and the need to book double appointments. Participants stressed the need to receive consistent information about their disease and medication from different professionals involved in their care. Different views were expressed regarding the ability to build rapport with a clinical pharmacist when compared to a GP. Input on study outcomes and design was provided by participants. **Conclusion**: participants in this session mentioned that a clinical pharmacy intervention after hospital discharge would be useful for their continuity of care. Plans are in place to continue to involve patients and the public in the write up, ethics and dissemination of the potential clinical pharmacy proposal.

## 1. Introduction

Myocardial infarction (MI) (a heart attack) is one of the most common types of circulatory diseases [1]. Statistics from the British Heart Foundation [1] show that heart and circulatory diseases cause more than a quarter of all deaths in the UK. In addition, there are more than 100,000 hospital admissions each year owing to heart attacks. Currently, there are around 7.4 million people living with heart disease in the UK, 3.9 million men and 3.5 million women. Patients are often discharged on around five medicines. These patients need continued support in primary care and help and advice about medicine use.

Patients who continue to take their medication after an MI have been found to have lower rates of recurrence, rehospitalisation and mortality compared with patients who discontinue [2,3,4,5,6,7,8]. However, adherence to secondary prevention medications by patients following an MI is often poor below 50% and declines markedly following initiation and steadily thereafter [8,9]. Thus interventions that are effective in improving levels of medication adherence after patient hospital discharge have the potential to reduce risks and improve clinical outcomes for patients [9]. Continued use of medicines is central to secondary prevention for patients following an MI. In particular, medicine-related problems and confusion can commonly arise following discharge. Furthermore, evidence shows that medicine optimisation is not optimal in primary care which could lead to consequences such as a second MI [10].

Most patients in the UK obtain their medicines from community pharmacies. Pharmacy is recognised as a resource that could contribute more effectively to health care and medicines use for people with long term illness [11,12,13]. However, there is only limited evidence to indicate how this might be achieved. In recent years pharmacy services in primary care that are part of the NHS contractual framework have become more diverse. For example medicines reviews such as the New Medicines Service (NMS) [11] have been introduced to address problems that patients experience with taking their medicines as prescribed and have been shown to identify problems and improve adherence to medicines when delivered by pharmacists [11]. Medicine reviews involve a pharmacist reviewing the patients’ use of their prescribed medications, making sure that they understand how their medications should be used, as well as the reason a medication has been prescribed. NMS is specifically aimed at service users who have recently been diagnosed with a long-term condition. The service intentionally focuses on improving medication adherence in order to enhance outcomes amongst service users who experience disproportionately poorer outcomes owing to their long-term condition.

Furthermore, important UK policy papers such as “Five Year Forward View” [12] and “The Murray Report” [13] have stressed the potential of a wider use of pharmacists in primary patient care. Evidence from NHS England [14] shows that the number of pharmacists based at general practices will increase. Although pharmacists roles and services have developed not all patients who urgently need these pharmacy services are aware of them as patients are not routinely referred to them.

This Patient and Public Involvement (PPI) focus group session in the present report was intended to provide piloted information of patient reported outcomes, perspectives and recommendations. In order to develop a proposal to involve clinical pharmacists. The proposal is trialling an intervention, delivered by pharmacists, for patients following an MI. The proposal will aim to propose a pathway of care to improve communication between all sectors involved in patient care including hospital, community and general practice (GP) services. The proposal will also include referral of patients into pharmacy services after hospital discharge.

## 2. Materials and Methods

This study is a community engaged research project involving a focus group format to obtain public perspective on utilization of clinical pharmacists to be able to develop a clinical pharmacy intervention in primary care. Although the study took the form of a focus group but it followed a patient and public involvement method and followed definitions and guidelines of INVOLVE (INVOLVE is a UK national advisory group that supports active public involvement in public health and social care research) [15]. PPI involvement in research is different from a research method, since members of public or patients take part actively in the design, conduct and management of a study. In addition they play an active role in the dissemination of findings. Such roles differ from participation as research subjects.

### Identification/Invitation

Contact with a large Cardiac Rehabilitation (CR) Centre in the West Midlands was made by the researcher (Z.J). The researcher gave a brief 10 min talk about the research study and asked patients and carers attending CR if they would be interested in taking part in a group discussion with other patients, carers and researchers on pharmacy services and pharmacy roles. Participants after the talk who showed interest signed up for the PPI discussion. An invitation leaflet explaining the study and the planned itinerary for the PPI discussion was handed out during the two-day recruitment.

The PPI discussion was held at the CR centre. A quiet room was booked. Patients who were invited to the PPI group discussion included adults (aged ≥ 18 years), who have been discharged from hospital after an MI, currently on secondary prevention medications and carers. The session was also an opportunity to recruit interested participants who would be willing to act as a full partner within the research team for the duration of the study and subsequent dissemination. This involvement will be fully costed in the research grant application, according to the National Institute for Health Research (NIHR) INVOLVE guidelines [15]. A topic guide was developed for the PPI discussion please refer to topic guide in Table 1.

The PPI discussion was recorded after the attendees provided consent. The discussion was transcribed verbatim, reviewed and checked by a member of the research team and a patient. The principal researcher reviewed the transcript analysis. A volunteer from the group was asked to review 10% of the transcript to ensure consistency.

Analysis: a thematic analysis was derived based on the focus group questions. Where individual responses were transcribed, coded and categorized, disagreement were resolved by two researchers regarding the categorization of statements from the focus group.

Ethical approval: This study was reviewed by the Research Design Service West Midlands (RDS WM) and approved as a public involvement event and therefore ethical approval was not sought. However, written consent was obtained from all participants to record the conversation and anonymised quotes are used for publication.

## 3. Results

Nine participants and three researchers attended the PPI discussion including five females and four males. Seven of the participants were patients who had experienced a cardiac event in the last three months and two were carers. Patients’ number of medicines that they were on ranged from 2 to 32 tablets with a mean of 11 tablets per day.

Key themes from the discussion are presented below with illustrative quotes and around the focus group questions:

1- What is your experience with your current pharmacy? Have you ever been to a pharmacy consultation such as a New Medicine Service? Have you ever been to a pharmacist at a general practice surgery?

*Theme 1—Patients’ experiences and knowledge about pharmacy services and primary care services.*

Different views were expressed regarding current pharmacy services. The majority of patients reported that pharmacy services such as medicines reviews and The NMS were unknown to them, enquiring about who should provide knowledge regarding these services. Participants also asked if patients should ask about these services and approach a pharmacist or is it the pharmacist’s responsibility. A lack of communication between patients and community pharmacists regarding such services was reported.

*P5 “How to contact a pharmacist? So, this is how it’s supposed to be but, a lot of it is not happening so therefore, what we think we should, people like you and, people unregistered, so not only like you, anyone, me, if I’m on medicine, especially if I’m on a new medicine, therefore I should know more about this service but I do not know and a lot of people do not know.”*

*P2 “… and a person, not used to going to pharmacists or doctors etc. Something new to me so, I find it very hard to communicate cause no-ones communicating to me first about my particulars. Am I making sense?”*

Different views were reported among pharmacy service users. One experience was reported as very useful and very helpful, while others perceived pharmacists to be very occupied and busy and just handing out medicines in a bag.

*P3 “I had a similar experience with my pharmacy, who said, this is a new medicine, step into the little cubicle and they, they very kindly explained it all before they let me leave but …”*

*P4 “It obviously depends on which pharmacy you go to.”*

*P2 “Yeah, so, I hear what we have is variations in how the services are being offered, possibly depends how busy these pharmacist are.” “I think, you know, the experiences often are, you know, the medicines being handed in a bag and there’s hardly any, any communication. Is that’s what’s happening in our opinion?”*

When pharmacy clinical services were delivered it was reported that pharmacists gave more time than doctors and did provide useful medication reviews.

*P7 “Mine are perfect, they pull me in every three months and check what medications I’ve been on and since the operations I haven’t actually been escorted into the little room, if you know what I mean, but, if I wanted to they would have check, they, they seem to have a bit more time to talk about than your doctor.”*

When asked whether they had an encounter with a pharmacist at a General Practitioner (GP) surgery to provide clinical services, patients reported that this was not something familiar and the familiar model was a pharmacy shop attached to a GP surgery. Only one patient reported an encounter with a GP practice-based pharmacist which they found to be a very useful review.

*P9 “Yes. I haven’t used it myself but, my husband had a review of his medicines in, at the GP practice, and it was a pharmacist. Now, I don’t know how often she comes in …”*

On the other hand a GP practice based nurse was a very familiar model, patients reported that the appointment with a nurse is usually very comprehensive, lasts for a significant amount of time when compared to a GP appointment and that there is no need to meet with a GP after seeing a nurse specialist.

*P1 “I see a specialist nurse on diabetes. And she, she manages … everything and … No need to see the GP. She’s had years of experience and the doctors can talking to them about it, the clinician will just say, talk to the specialist nurse, I’m very happy with the arrangement.”*

*P1 “My appointment time with my specialist nurse are half an hour, 30 min … just go right through the whole thing.”*

Patients reported that they would value a similar model with a specialist pharmacist, where a comprehensive review would help provide reassurance on the medication they were prescribed.

*P3 “Yes, I’m in Lawton as well and they have specialist nurses. I would think that the doctors would be delighted to have a pharmacist to take over certain duties such as this and taking away from them, yeah.”*

Patients reported that appointments with GPs were very limited around 10 minutes maximum and that sometimes they would have to book a double appointment to be able to discuss problems they have with their medicines and disease.

*P4 “Yeah, yeah, I mean, my doctor’s very nice, there’s another one I won’t see but you do sometimes, you know, why they don’t give you longer, erm, waste of time, I know.”*

*P9 “I think sometimes can you not book double appointments now?”*

2- What do you think about your discharge summary been sent to your pharmacist?

When asked if patients would be happy to share their hospital discharge summary with a pharmacist, the majority showed willingness and reported that they would trust a pharmacist with this summary. In addition patients reported that it would be useful for them to be contacted by a pharmacist after discharge regarding their medications.

*P9 “with the pharmacist as well and it takes off from the hospital and it takes off the waiting time for discharge that you can have as well, yes it is a good idea.”*

*P2 “Yeah, erm, and, you know, some of them (pharmacists), even if they’re not in a prescribing capacity, I think that review could be done in practice, you know, or in the community so, that’s another way of, you know, helping with medicines.”*

3- Has a pharmacist ever visited you at your home?

Discussions also revolved around having a pharmacist visit at home and some patients mentioned that this was not familiar for them while others reported few isolated occasions where a pharmacist was present at a care home.

*P3 “Sorry, yes, my mother-in-law actually did have a pharmacist visit her with the carers to go through her drugs.”*

*Theme 2 and 3—Medicines knowledge and a pharmacist role.*

Patients expressed the need for further explanation and knowledge regarding the prescribed medication after a heart attack this was due to the fact that the drugs are something new to them, that they are not experts in medicines and find it difficult to even pronounce the names and of the drugs. The discharge summary was also mentioned to be difficult to understand from a layperson’s point of view. Patients reported that they needed reinforcement of the messages regarding their medication as it was difficult to recall information given during hospital stay.

*P7 “I also find, if they asked me what medication, the language, the pronunciation of the words, what I would find stressful as well, very hard to explain.”*

*P8 “when I came out the hospital, I couldn’t tell you anything what they told me, you know, to go to the GP, about your medications, this that and the other so, it would be, that’s something that could do with a bit help further …”*

Patients reported sometimes receiving conflicting messages from different Health Care Professionals (HCPs) involved in their care regarding the timings to take medicines and instructions about the medicines; which left patients sometimes confused. However, after having a longer discussion with a pharmacist at cardiac rehabilitation and a better understanding helped make things clearer. This shows the importance of a medication review in primary care.

*P2 “Yes, possibly, down to our understanding, just as a small point, understanding the discharge letters so, so, I was seeing my consultant and I say, why He said, oh, you’re having eight a day for breakfast, I said, no, it’s more. I said, oh, okay, and then I get my pharmacist and he says, do not take those at this point.”*

*P2 “And then he said, today, actually, let me explain, cause I said well why, now is it the rate of absorption with the statin …”*

Patients expressed that they perceive the current pharmacist role to be a supplier and a service provider more than a person to go to for advice. Patients agreed that they forget that pharmacists are experts in medicine and that pharmacists have extensive medication knowledge and experience. Some patients expressed that they do visit a pharmacist abroad for advice instead of a doctor, also they would go to a UK—based pharmacy for minor ailments. However, in the UK the first port of call would be the doctor.

*P7 “So, I haven’t had a major experience with the pharmacist. When I’ve had to go in, I’ve seen pharmacies as a service provider.”*

Pharmacists in the community are perceived as incredibly busy, should not be bothered, isolated behind the counter, with limited interactions with patients.

*P3 “I think when we’re told, when we go to the pharmacist and they’re incredibly busy …”*

*P1 “Yeah, I think that’s what’s showing, that a pharmacist is behind the counter, isolated somewhere, you don’t even know which ones the pharmacist and they don’t come to you, they don’t actually come out and talk to you.”*

Patients reported a lack of knowledge regarding public health services offered by community pharmacies in the UK such as flu vaccination, blood pressure measurement, travel health and even prescribing rights for independent prescribing pharmacists; this was referred to as not advertised or promoted. Patients reported the need for pharmacy services to be made more public and to raise awareness regarding available services.

*P2 “It’s the, it’s the reality that, you know, but I think it’s not reaching out, it’s not being promoted and we, you know, patients don’t know, people don’t know it’s, basically, it’s not advertised.”*

Patients expressed that if a pharmacist was responsible for their medicines then this could relieve the current pressures on GPs.

*P7 “If the pharmacist was looking after the medication you wouldn’t even need to go to your doctor to get a repeat prescription would you, the pharmacy could do it for you …”*

Recognition of pharmacists’ knowledge and capabilities as highly trained HCPs was highlighted in parts of the discussion.

4- How do you build rapport with your pharmacist, general practitioner?

*Theme 4—Building Rapport with healthcare professionals.*

Patients expressed the need to build rapport with their current doctor and reported that continuity of care was important to them (seeing the same doctor).

*P7 “I don’t have a problem, I have to say. Normally, unless I need an instant appointment or something, an emergency, I’ll wait for an appointment with my doctor in preference to see him. If I need a locum, or somebody else in the practice, if it’s urgent enough I will but, yes, do you a build up a rapport with your doctor, yes.”*

Different views were expressed when asked about building rapport with a pharmacist. Some patients reported that they could build rapport with a pharmacist. One patient reported that he had a good relationship with his pharmacists during medication reviews. While others mentioned that pharmacists are busy especially when there are many waiting patients and queues, in addition to the presence of locum pharmacists and therefore found it difficult to build rapport.

*P2 “Well, we did have one for about three or four years and he was excellent. I’ve got six, erm, serious conditions, chronic conditions, that’s obviously why I started having reviews and, so, we did, I built up a rapport with this pharmacist because he knew exactly the medicine I needed. We talked about it and that’s why, you know, so, it can work but, at the moment, we’re getting changes sort of every other month …”*

*P1 “I’ve turned up and they’ve been queued out the door, there’s been about 20 in the queue. So, how that person’s going to afford that time to do that, is another question.”*

Participants were provided with a diagram of the study and also a list of the outcomes to achieve for the potential research project that this PPI session was designed to inform. Participants listed the outcomes in order of what they felt is most important for their care (Table 2). The following outcomes are listed in order of importance to patients and members of the public. The outcomes and their order of preference will be taken into consideration for the design of the potential clinical pharmacy proposal.

## 4. Discussion

Patients reported that the potential study could help them after a cardiac event where a pharmacist as part of a multidisciplinary team could provide follow up on medicine use. The care pathway in the potential study will involve pharmacists in different sectors in primary care, and links with secondary care. Pharmacists will provide education, in addition to treatment optimization. Patients welcomed the idea of sharing their discharge summary with a pharmacist. They were happy to be contacted by a pharmacist as this could provide an opportunity to reinforce important information regarding medication related problems and issues. Such findings are in-line with our previous study [16] where community pharmacists in London provided telephone and face to face consultations for patients after an MI. Both adherence and clinical outcomes such as lipids and blood pressure control were improved. Patients in this feasibility study in interviews expressed satisfaction with pharmacy consultations [16]. Previous studies report the importance of capturing patients’ perspectives early in service design to be able to achieve high quality care [17,18].

Patients reported that a GP practice-based pharmacist should be experienced and approachable. Patients also expressed the importance of having consistent information and key messages about their disease and medication and reported an emphasis on effective communication skills for healthcare professionals (HCPs) in general for doctors, pharmacists and nurses. Also, the need for more time in current GP consultations was emphasised. Consultations were reported as being very short and that this could be resolved if support was available from other HCPs for example pharmacists. Studies show the need to involve patients in their decision making process around their disease and medication. This is to be able to build effective patient–doctor relationships which could affect clinical outcomes [19].

Further areas of involvement of patients in this future study will be during the operation of the study. Patients will be involved in the review of patient information and consent forms during the ethics application. As evidence emphasizes the importance of having readable and understandable written documents for an informed consent [20]. Patients will advise on the dissemination of findings. Patients’ perspectives will also be sought on interpretation of results and study findings. The participants were informed that they will be updated in writing about the application proposal outcome and if successful on the findings of the study. Participants were informed that a poster would be designed for a pharmacy conference from this session to showcase the importance of PPI in research design and development.

### 4.1. Strengths and Limitations

This patient and public involvement session provided an insight on service users experiences and views on current clinical pharmacy services and roles. Their advice and views will help shape and design a clinical pharmacy intervention for patients after a cardiac event. A member of the public will contribute to the project as a co-applicant for the research grant and we have recruited advisors to sit on the steering group for the grant application. A qualitative method was followed; however, data saturation was not sought. The PPI discussion was conducted at one CR centre in the West Midlands and included a small sample size of participants. Therefore, the findings are not generalizable to a wider UK population.

### 4.2. Future Plans

Patients in this PPI session felt it would be useful to have a pharmacist based at a GP surgery and would be happy to be referred to when needed similar to a GP practice based nurse which they found from experience to be a good model of care. Such model of care, including specialist nurses in GP practices, has shown safe and effective practice with positive outcomes and patient satisfaction [17]. This is supported by strong evidence showing nurse-led care substituting physician roles and achieving significant results in outcomes such as reduced hospital admission and mortality, in various primary care models and in various chronic disease conditions [21].

Patient and public advice will help develop the design of a clinical pharmacy intervention with a new pathway of care for patients after a cardiac event. With the aim to improve medicine optimisation and safe medicine use. Funding will be sought from NIHR for the operation and evaluation of the service.

## 5. Conclusions

Four key themes emerged from this focus group “experiences with pharmacy and primary care services, medicines knowledge, the pharmacist role and building rapport with healthcare professionals,” In addition the participants helped in prioritizing outcomes for an intervention, these themes will help in designing the proposed clinical pharmacy intervention. Plans are to continue to involve patients and public in the input into the potential clinical intervention design, conduct and dissemination of results.

## Figures and Tables

**Table 1:** Topic Guide for Patient and Public Involvement (PPI) focus group discussion.

Questions for the Discussion Session
Discussions included a summary of what the research is about and discussions around patients experiences and knowledge about pharmacy services and primary care services.
Also discussions around what problems patients face with their medicines, where do they go for resources and information, concerns and beliefs regarding their disease and medication.
The session was around how important patients perceive pharmacy services to be and to understand which of the study outcomes are most important to patients.
Questions included: What is your experience with your current pharmacy? Have you ever been to a pharmacy consultation such as a New Medicine Service? Could you share your experience? Have you ever been to a pharmacist at a general practice surgery? Could you please share your experience? How do you build rapport with your pharmacist, general practitioner? What do you think about your discharge summary been sent to your pharmacist? Has a pharmacist ever visited you at your home?

**Table 2:** List of outcomes prioritized by community members.

Outcomes Listed in Order
Not having another heart attack
Not going backto hospital
Wellbeing
Knowledge about medicines
Medication adherence and usage
